# Expression of Mitochondrial Long Non-Coding RNAs, MDL1 and MDL1AS, Are Good Prognostic and/or Diagnostic Biomarkers for Several Cancers, Including Colorectal Cancer

**DOI:** 10.3390/cancers16050960

**Published:** 2024-02-27

**Authors:** Pablo Garrido, Adrián Casas-Benito, Ignacio M. Larrayoz, Judit Narro-Íñiguez, Susana Rubio-Mediavilla, Enrique Zozaya, Alfonso Martín-Carnicero, Alfredo Martínez

**Affiliations:** 1 Angiogenesis Group, Oncology Area, Center for Biomedical Research of La Rioja (CIBIR), 26006 Logroño, Spain; pgarridor@riojasalud.es (P.G.); acasas@riojasalud.es (A.C.-B.); jnarro@riojasalud.es (J.N.-Í.); 2 Department of Nursing, Biomarkers, Artificial Intelligence and Signaling (BIAS), University of La Rioja, 26004 Logroño, Spain; ignacio.larrayoz@unirioja.es; 3 Pathology Service, Hospital San Pedro, 26006 Logroño, Spain; srubiom@riojasalud.es; 4 Pathology Service, Hospital de Calahorra, 26500 Calahorra, Spain; ezozaya@riojasalud.es; 5 Medical Oncology Department, Hospital San Pedro, 26006 Logroño, Spain; amcarnicero@riojasalud.es

**Keywords:** rectal cancer, breast cancer, long non-coding RNAs, mitochondria, oxidative phosphorylation, growth, migration

## Abstract

**Simple Summary:**

Personalized cancer medicine is based on the right classification of patients that can, then, be treated according to their specific characteristics. This is accomplished by the use of biological markers, so the identification of reliable biomarkers is a primary goal of clinical research. Here, we present two RNA molecules (named MDL1 and MDL1AS) that are generated in the mitochondria and have been heretofore neglected due to a glitch in the official human genome. Both molecules are good prognostic biomarkers in rectal cancer, meaning that their expression can predict which patients will survive more than 5 years after treatment. In addition, MDL1AS is also a good diagnostic biomarker (can distinguish people with/without the disease) for diverse cancers, including those of the colon, rectum, breast, and larynx. Experiments in cancer cells in culture show that these RNAs regulate several hallmarks of cancer, such as mitochondrial function, cell growth, and migration.

**Abstract:**

Non-coding RNAs provide new opportunities to identify biomarkers that properly classify cancer patients. Here, we study the biomarker status of the mitochondrial long non-coding RNAs, MDL1 and MDL1AS. Expression of these genes was studied in public transcriptomic databases. In addition, a cohort of 69 locally advanced rectal cancer (LARC) patients with a follow-up of more than 5 years was used to determine the prognostic value of these markers. Furthermore, cell lines of colorectal (HCT116) and breast (MDA-MB-231) carcinoma were employed to study the effects of downregulating MDL1AS in vitro. Expression of MDL1AS (but not MDL1) was significantly different in tumor cells than in the surrounding tissue in a tumor-type-specific context. Both MDL1 and MDL1AS were accurate biomarkers for the 5-year survival of LARC patients (*p* = 0.040 and *p* = 0.007, respectively) with promising areas under the curve in the ROC analyses (0.820 and 0.930, respectively). MDL1AS downregulation reduced mitochondrial respiration in both cell lines. Furthermore, this downregulation produced a decrease in growth and migration on colorectal cells, but the reverse effects on breast cancer cells. In summary, MDL1 and MDL1AS can be used as reliable prognostic biomarkers of LARC, and MDL1AS expression provides relevant information on the diagnosis of different cancers.

## 1. Introduction

Cancer is already the main cause of death in the world. Colorectal cancer is the third most common malignancy, with an incidence of 1.9 million people a year, representing 10% of all cancers, and has an estimated mortality of 935,000 cases per year, 9.4% of all cancer deaths [1]. The majority of colorectal cancers have their origin in sporadic mutations, although there is a small hereditary group [2]. The more common treatment for advanced colorectal cancer consists of multimodal management, which includes surgery, adjuvant, and neoadjuvant therapies [3].

In order to diagnose and follow up cancer progression and treatment, biomarkers are paramount. A biomarker is a molecule, organism, or sequence of genetic material that can be used to measure the presence or absence of a disease, its progression, or its response to treatment. A biomarker must have several characteristics, such as high sensitivity, specificity, and safety [4]. In colon cancer, the alterations produced by chromosomal instability (CIN), microsatellite instability (MSI), or the CpG island methylated phenotype (CIMP), produce changes in DNA, RNA, proteins, and metabolites that can be measured in the tumor, blood, or stool. These changes can be used as diagnostic or progression biomarkers [5]. These biomarkers can be used to predict the outcome of a therapeutic intervention or to provide information on the response to a treatment [6]. Despite biomarkers being the path to improve cancer survival through personalized treatments, the number of clinically relevant biomarkers in colon cancer is rather low. The usefulness of biomarkers is exemplified in patients with elevated MSI. For these patients, treatment with 5-fluorouracil is not effective, whereas oxaliplatin provides a working therapeutic option [6]. Another example is the mutations in RAS, KRAS, or BRAF, which act as biomarkers of response to treatment, since tumors with mutations in these genes present resistance to anti-EGFR treatment with cetuximab [7].

Recently, biomarkers based on long non-coding RNAs (lncRNAs) have been shown to regulate different cancer cell functions such as cell proliferation, migration, angiogenesis, cell death, and metastasis. Thus, some of them can be used as biomarkers of the disease [8]. For example, ZEB1-AS1, FAM83H-AS1, LINC01296, and LINC01234 correlate with clinicopathological parameters and survival of colorectal cancer patients, and high expression of ZEB1-AS1 is associated with poor survival prognosis [9]. 

In cancer cells, mitochondria are critical organelles, which are involved in tumor proliferation, survival, metastasis, and drug resistance [10]. Tumor cells use glycolysis to produce energy in detriment of oxidative phosphorylation. This characteristic was described by Otto Warburg a hundred years ago, and is known as the “Warburg effect” [11,12]. These interconnected mechanisms stimulate tumor cell invasion, metastasis, angiogenesis, and immunosuppression [13]. Mitochondria have their own double-stranded circular genome, with a length of 16,569 bp. The heavy chain codes for 12 proteins, 2 rRNAs, and 14 tRNAs, while the light chain codes for a single protein (ND6) and 8 tRNA [14]. In the mitochondrial genome, there are two replication origins for the heavy strand, called Heavy-strand promoter 1 and 2 (HSP1, HSP2), and one for the light strand, called light-strand promoter (LSP), located in the D-loop region [14]. This region is important in some diseases, including cancer, because this area can accumulate a large number of mutations that may impact cancer progression [15]. 

In the mitochondrial genome, we also find non-coding RNAs (ncRNAs), such as several micro-(miRNAs), circular-(circRNAs), and long non-coding (lncRNAs) RNAs. Examples of the latter include lncND5, lncND6, and lncCytb, which are encoded on the antisense strand of the ND5, ND6, and Cybt genes, respectively [16]. Some circRNAs in the mitochondrial genome are mc-COX2, mecciND1, and mecciND5 in the heavy strand, and circRNA SCAR in the light strand [17], and thousands of miRNAs such as hsa-mitosR-L-DL [18] or mito-ncR-805 [19].

Some of these ncRNAs are related to different diseases. For instance, Mitochondrially Encoded Long Non-Coding Cardiac Associated RNA (MT-LIPCAR) acts as a biomarker in heart disease [20]. There are lncRNAs whose expression or localization are altered in cancer, and they are thought to play a role in the development of the disease, although their functions are currently under investigation. In an in vitro model, lncCytb has been shown to localize in the mitochondria in normal cells, whereas it translocates to the nucleus in tumor cells [21], although some authors have suggested that lncCytb, lncND5, and lncND6 play a role stabilizing Cytb, ND5, and ND6 by regulating their expression [22]. Other mitochondrial lncRNAs are sense non-coding mitochondrial RNAs (SncmtRNA) and the two antisense ASncmtRNA-1/2. These lncRNAs are transcribed at the 16S region; they play a role in the cell cycle, and have an aberrant expression in different tumors [23]. 

Two more mitochondrial lncRNAs with variable expression between normal and tumor cells were discovered in 2018, namely, Mitochondrial D-loop 1 (MDL1) and Mitochondrial D-loop 1 antisense (MDL1AS) [24]. As their name indicates, these lncRNAs are coded in the D-loop region; MDL1 starts at position 15,954 and ends at 576 of the heavy strand, whereas MDL1AS starts at 16,024 and ends at 407 of the light strand [24]. MDL1 has been shown to translocate into the nucleus where it interacts with a network of nuclear genes, including p53, whose function is compromised as a consequence [25]. The translocation of MDL1AS to the nucleus has been also proposed in a recent preprint [26], although no specific function has been described for this lncRNA. Since not much is known about these mitochondrial lncRNAs, the aim of this study was to better understand their role in cancer biology and their biomarker value in several cancers, including rectal cancer. 

## 2. Materials and Methods

### 2.1. Data Mining

To investigate the expression of MDL1 and MDL1AS in existing transcriptomic databases, files were obtained from the National Center for Biotechnology Information (NCBI) website by searching for projects that provide transcriptomic data from tumors and corresponding normal adjacent tissues. The fastq archives were downloaded from BioProjects: PRJNA482141, PRJNA510105, PRJNA552068, PRJNA684607, PRJNA760779, and PRJNA434883. These include cases from colon, rectal, breast, or laryngeal cancers. 

### 2.2. Mitochondrial Reference Genome Edition and Bioinformatics

Since MDL1 and MDL1AS are located over the replication origin of the mitochondrial genome, their expression cannot be mapped on the regular human genome, which offers a lineal model of the mitochondrial circular genome interrupted precisely at the heavy strand’s replication origin. Thus, a mitochondrial reference genome was created using MITOS2 webServer, based on the suggestions offered by Gao et al. [24]. Sequence alignments were performed with STAR-2.7.7a and they were quantified with featureCounts v2.0.0. Finally, reads per kilobase of exon model per million mapped reads (RPKM) was used to normalize results. The data obtained were analyzed with false discovery rate (FDR) protocols to identify those mitochondrial genes whose expression varies significantly in relation to relevant clinical criteria. Sequence alignment and quantification were performed under the R environment.

### 2.3. Patients

Initially, 92 patients that were treated for LARC between the years 1998 and 2017 at Hospital San Pedro (Logroño, La Rioja, Spain) and Hospital de Calahorra (La Rioja, Spain) were preselected, but 23 of them had to be excluded because their paraffin blocks containing the initial tumor biopsy were deteriorated. As a result, a total of 69 patients were included and analyzed. All procedures were approved by the Medical Research Ethics Committee of La Rioja (CEICLAR, protocol number PI-129, 31 July 2013) and all patients or their families signed the informed consent before inclusion. The treatment protocol and clinical characteristics of this cohort have been previously published [27]. Briefly, all patients were treated with neoadjuvant chemoradiotherapy for five weeks. Radiotherapy consisted of 44–45 Gy delivered in fractions of 1.8 Gy per day, five days per week, and fluorouracil, administered as monotherapy as oral capecitabine (875 mg/m^2^/12 h every day) or given in a continuous intravenous infusion (225 mg/m^2^/day), or combined with oxaliplatin in the scheme known as FOLFOX-6 (5FU bolus 400 mg/m^2^), leucovorin (400 mg/m^2^), oxaplatin (85 mg/m^2^), and 5FUci (240 mg/m^2^ every two weeks). Total mesorectal excision surgery was performed 7–9 weeks after completion of chemoradiotherapy. After surgery, all patients were offered adjuvant chemotherapy, depending on the pathological stage.

Paraffin blocks from the initial tumor diagnosis of all patients were retrieved from the pathology departments of both hospitals. Two 4 µm thick sections were obtained from each block, and total RNA was isolated with the High Pure FFPET RNA Isolation Kit (Roche, Sant Cugat del Valles, Spain). cDNA was obtained and subjected to next-generation sequencing (NGS) using Illumina protocols and the HiSeq 1500 platform (Illumina, San Diego, CA, USA), as described [28]. 

### 2.4. Cell Lines and Culture

The colorectal carcinoma cell line HCT116 and the breast adenocarcinoma cell lines MCF7 and MDA-MB-231 were obtained from the American Tissue Culture Collection (ATCC, Manassas, VA, USA), cultured in DMEM medium (Corning, New York, NY, USA) supplemented with 10% fetal bovine serum (HyClone, Logan, UT, USA), and maintained in a 37 °C environment, with an atmosphere containing 5% CO_2_ and 85% humidity.

### 2.5. Gene Downregulation

To study the physiological functions of MDL1AS, its expression was modulated with double-stranded interfering RNAs (DsiRNAs), as described [29]. All DsiRNAs were synthetized by Integrated DNA Technologies (Coralville, IA, USA) at 10 nmol scale (Table 1). They were diluted in RNAse-free water to a final concentration of 100 µM. For transfection, HCT116 (150,000–250,000 cells per well), MCF7, and MDA-MB-231 (50,000–100,000 cells per well) cells were seeded in six-well plates and transfected with Lipofectamine RNAiMAX (Thermo Fisher, Waltham, MA, USA), according to manufacturer’s instructions, in serum-free medium and using DsiRNA at a concentration of 10 nM.

### 2.6. RNA Extraction and qRT-PCR

RNA extraction from cell cultures was performed using TRIzol (Invitrogen, Carlsbad, CA, USA) and the RNeasy Micro Kit (Quiagen, Germantown, MD, USA) according to the manufacturer’s instructions and as previously described [30]. cDNAs was mixed with NZYSupreme qPCR Green Master Mix (NZYTech, Lisboa, Portugal) and analyzed in the QuantStudio 5 Real-Time PCR thermocycler (Applied Biosystems, Norwalk, CT, USA). Cycling conditions were 95 °C 10 min, followed by 40 cycles of 95 °C 15 sec and 60 °C 1 min. At the end, a dissociation curve was implemented from 60 to 95 °C. The PCR primers (Table 2) were added at a final concentration of 0.3 µM. A standard curve was included in every plate to obtain a relative quantification of all genes. QuantStudio Design & Analysis Software v1.5.1 (Applied Biosystems) was used to analyze the data. ND1 and GAPDH were used for normalization.

### 2.7. Mitochondrial Metabolism

Mitochondrial metabolism was measured with the Seahorse XFe24 Analyzer (Agilent, Santa Clara, CA, USA) using the Mito Stress Test, following the manufacturer´s instructions. Briefly, 30,000 HCT-116 or 20,000 MDA-MB-231 cells were seeded per well in Seahorse´s 24-well plates, and the assay cartridge was hydrated and kept in an incubator at 37 °C with no addition of CO_2_. The next day, cells were washed with DMEM medium, pH 7.4, supplemented with 10 mM glucose, 1 mM pyruvate, and 2 mM L-glutamine (Agilent), and the same medium was added for a 45 min incubation at 37 °C in the absence of CO_2_. Modulating compounds used in the assay were: 1.5 µM oligomicin, 0.5 µM rotenone/antimycin A (Agilent), and 0.5 µM carbonyl cyanide 4-(trifluoromethoxy) phenylhydrazone (FCCP, Sigma, Saint Louis, MO, USA). Results were analyzed using Seahorse Wave Software v2.6.3. Data normalization was achieved by calculating total protein concentration by BCA assay (Thermo Fisher) at the end of the procedure, following the manufacturer’s instructions. 

### 2.8. Proliferation Assay

To measure cell proliferation, the MTS technique was used as reported [31]. Briefly, HCT-116 (3000 cells/well) or MDA-MB-231 (2000 cells/well) cells were seeded in 96-well plates (Falcon). Half of the wells were transfected with the DsiRNA, as explained above. Cell density was estimated at 24, 48, 72, and 96 h after seeding the cells. To achieve this, 20 µL CellTiter 96^®^ AQueous One Solution Cell Proliferation Assay (Promega, Madison, WI, USA) were added per well, incubated for 4 h, and the final absorbance was read at 490 nm using a POLARstar Omega (BMG Labtech, Ortengerb, Germany) plate reader.

Since the MTS technique is based on mitochondrial activity [32] and we found defects in mitochondrial function following downregulation of MDL1AS, we used an alternative mitochondria-independent method to establish cell number. For this, 75,000 cells were seeded per well in 12-well plates, one plate for control and one plate for transfected cells. Three wells of each plate were analyzed in duplicates at 24, 48, 72, and 96 h after seeding, using Trypan Blue (Gibco, Billings, MT, USA) staining, and viable cells were counted directly (TC20 Automated Cell Counter, BioRad, Hercules, CA, USA). 

### 2.9. Migration Assay

To investigate whether MDL1AS downregulation impacted on cell migration, a cell scratch assay was performed. Cells were grown in 6-well plates until they formed a continuous monolayer. At this time, a wound was performed with a 200 µL micropipette tip. Specific areas were labeled and photographed at 0, 24, 48, and 72 h after wounding using a DMI4000B inverted microscope (Leica Microsystems, Wetzlar, Germany) equipped with a DFC300 Fx digital camera (Leica). The surface that was not occupied by cells was subsequently analyzed and quantified using ImageJ v1.54h (NIH, Bethesda, MD, USA).

### 2.10. Statistical Analysis

The characteristics of the patients were described by means and standard deviations, medians and interquartile ranges, or frequencies and percentages, depending on the normality and nature of the variables.

Comparisons between groups were performed by ANOVA followed by Sidak´s multiple comparisons test. Patients were classified according to their time of survival after diagnosis into survivors (≥5 years) and nonsurvivors (<5 years). The Kaplan–Meier method was used for the analysis of survival, and the log-rank test and the Cox proportional hazards model were used to compare the survival between groups. Receiver operating characteristic (ROC) curves were constructed, and the sensitivity and specificity, as well as the area under the curve (AUC), of the biomarkers were calculated. The Youden Index methodology was performed to find optimal cut-off values [33]. Differences were considered statistically significant for a two-tailed test when *p* < 0.05. Version 20.0 of SPSS Inc. (Chicago, IL, USA) was used for all statistical analyses.

## 3. Results

### 3.1. MDL1AS Expression Levels in Different Cancers

**Figure 1:**
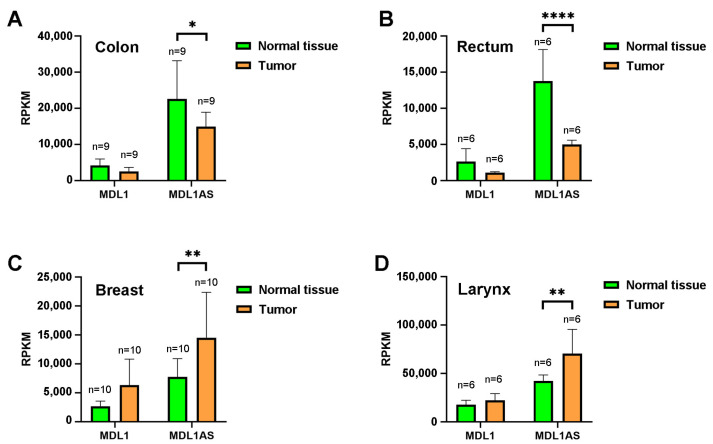
Expression of MDL1 and MDL1AS through data mining of public transcriptomic databases representing cancers of the colon (**A**), rectum (**B**), breast (**C**), and larynx (**D**). The expression in the tumor (orange) is compared with the expression in the surrounding normal tissue (green). RPKM: Reads per kilobase of exon model per million mapped reads. Each bar represents the mean ± standard deviation (SD) of each group. The number of subjects in each group are indicated. Statistical analysis: one-way ANOVA followed by Sidak´s multiple comparisons test. *: *p* < 0.05; **: *p* < 0.01; ****: *p* < 0.0001.

We began our study by analyzing the levels of MDL1 and MDL1AS in a variety of tumor specimens compared with the expression in the surrounding normal-looking tissue. To perform this, we used data mining from existing public repositories of transcriptomic analyses for different cancers. No significant differences were found for the expression of MDL1, but the expression of MDL1AS was different between tumors and adjacent normal tissues (Figure 1). Interestingly, in rectal and colon cancers, MDL1AS expression was significantly lower in the tumor than in the normal tissue surrounding the tumor (Figure 1A,B), whereas in breast and laryngeal cancers we found the reverse pattern (Figure 1C,D). 

### 3.2. Patient Characteristics

The clinical study sample consisted of 69 LARC patients, 20 (28.99%) women and 49 (70.01%) men. The median (Q1–Q3) age was 62 (30–79) years. The median follow-up was 10.7 (3.72–13.67) years. Sixty-five patients (94.2%) had a clinical stage (cTNM) ≥ T3N0. The median distance from the tumor to the anal margin was 6.39 (1–15) cm, and the majority were located in the middle and lower rectum (88.6%). Thirty-eight (55%) patients had a history of smoking (active smokers or former smokers). The median preoperative CEA level was 10.88 (0.6–77.5) ng/mL. At the molecular level, 9 (13%) patients had microsatellite instability (MSI) and 16 (23.5%) patients had KRAS mutations (Table 3).

Regarding the pathological response to neoadjuvant treatment, 28 (40.57%) patients had a pathological stage (ypTNM) ≥ T3N0. The tumoral response grading, according to the modified Ryan classification system recommended by the College of American Pathologists [34], could be assessed in 67 (97.1%) patients. Of these, 25 (37.13%) patients presented TRG0-1 (7 TRG0), while 42 (62.67%) presented TRG2-3 (13 TRG3). Seventeen (25%) patients had lymph node involvement, with an average of 8.43 (0–17) isolated nodes and a mean of 0.84 (0–7) infiltrated nodes (Table 3).

In a previous study, the transcriptomic profile of these patients was analyzed, looking for potential prognostic or diagnostic biomarkers, based on differential gene expression. None of the genes had any biomarker potential. 

### 3.3. Expression of MDL1 and MDL1AS in LARC Patients Predict 5-Year Survival

Given the differential expression of MDL1AS between rectal cancer cells and the surrounding normal tissue (Figure 1A) and that the differences in levels of expression for this lncRNA were almost significant in patients with long survival when compared to those with lower survival (*p* = 0.053, Table 1), we decided to study more deeply the connections between the expression of MDL1/MDL1AS and LARC patient survival.

**Figure 2:**
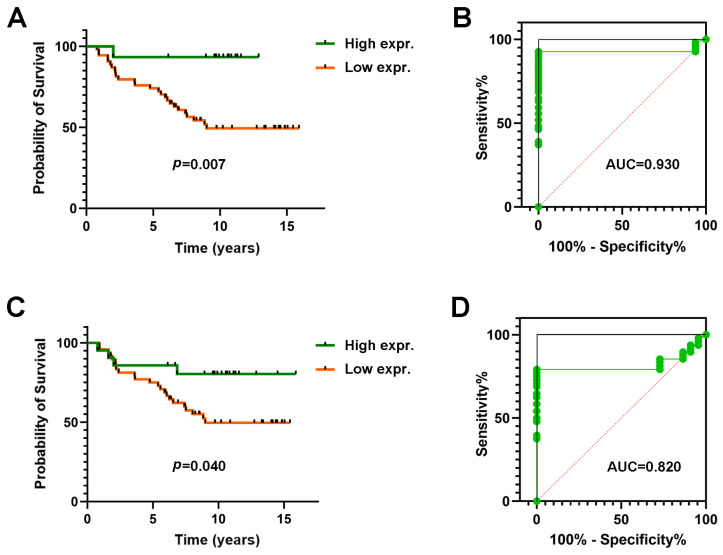
Kaplan–Meier graphs representing lncRNA-expression-dependent patient survival (**A**,**C**) and ROC curves for predicting 5-year survival (**B**,**D**) for MDL1AS (**A**,**B**) and MDL1 (**C**,**D**). Statistical analysis: Mantel–Cox test. AUC = area under the curve.

To determine the best way to classify patients, the Youden Index was calculated for MDL1AS expression, finding an optimal cut-off value at 1980 RPKM, which showed a survival advantage for patients with a higher level of MDL1AS expression (Mantel–Cox test *p* = 0.007, Figure 2A). An ROC analysis showed an AUC = 0.930 (95% CI 0.863–0.997; *p* < 0.0001) with a sensitivity of 92.6% (82.4–97.1) and a specificity of 100% (85.1–100) (Figure 2B).

Interestingly, expression of MDL1 (optimal cut-off value = 2382 RPKM) was also able to significantly predict patient survival, although with less efficacy than MDL1AS (Mantel–Cox test *p* = 0.040, Figure 2C). The ROC analysis for MDL1 had an AUC = 0.820 (95% CI 0.718–0.923; *p* < 0.0001) with a sensitivity of 79.2% (65.7–82.3) and a specificity of 100% (85.1–100) (Figure 2D).

When patients were separated by their MDL1AS expression, no statistically significant differences, other than the expression of MDL1 and MDL1AS, were encountered for other factors (Table 4), indicating that MDL1AS expression may be an independent biomarker. 

### 3.4. MDL1AS Expression Can Be Downregulated by Specific DsiRNA Sequences

**Figure 3:**
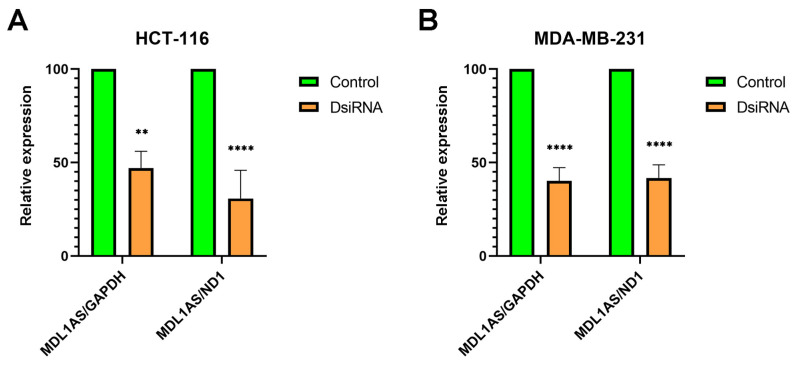
Inhibitory efficiency of the DsiRNAs in reducing MDL1AS expression in colon (**A**) and breast (**B**) cancer cell lines. Cell lines were exposed to the DsiRNAs (or to vehicle in controls) and their total RNA was purified. Expression of MDL1AS was analyzed through qRT-PCR and the values were relativized to the expression in untransfected cells (control, green). Statistical analysis: one-way ANOVA followed by Sidak´s multiple comparisons test. **: *p* < 0.01; ****: *p* < 0.0001.

To better understand the physiological implications of MDL1AS expression in different cancers, we investigated the function of this lncRNA in cancer cells, using cells from the colon (HCT-116) and the breast (MCF7 and MDA-MB-231) that clinically showed different behaviors (Figure 1). To modulate MDL1AS expression, we used the DsiRNA technique. Several sequences were tested (Table 1) and all of them were effective in reducing MDL1AS expression, with MDL1AS.3 producing the best results, as tested by qRT-PCR, in colon cell lines, whereas MDL1AS.2 was better for breast cancer cells (Figure 3). Interestingly, this reduction was confirmed both when MDL1AS expression was relativized to the nuclear gene GAPDH or to the mitochondrial gene ND1 (Figure 3A,B), suggesting that the DsiRNAs affected only the MDL1AS gene but not the rest of the mitochondrial genes. The reduction in MDL1AS expression was time-dependent and the maximum effect was observed at 48 h post-transfection for MDA-MB-231 and at 72 h for HCT-116. Unexpectedly, all MCF7 cells died after transfection.

### 3.5. MDL1AS Downregulation Reduces Mitochondrial Respiration Parameters 

**Figure 4:**
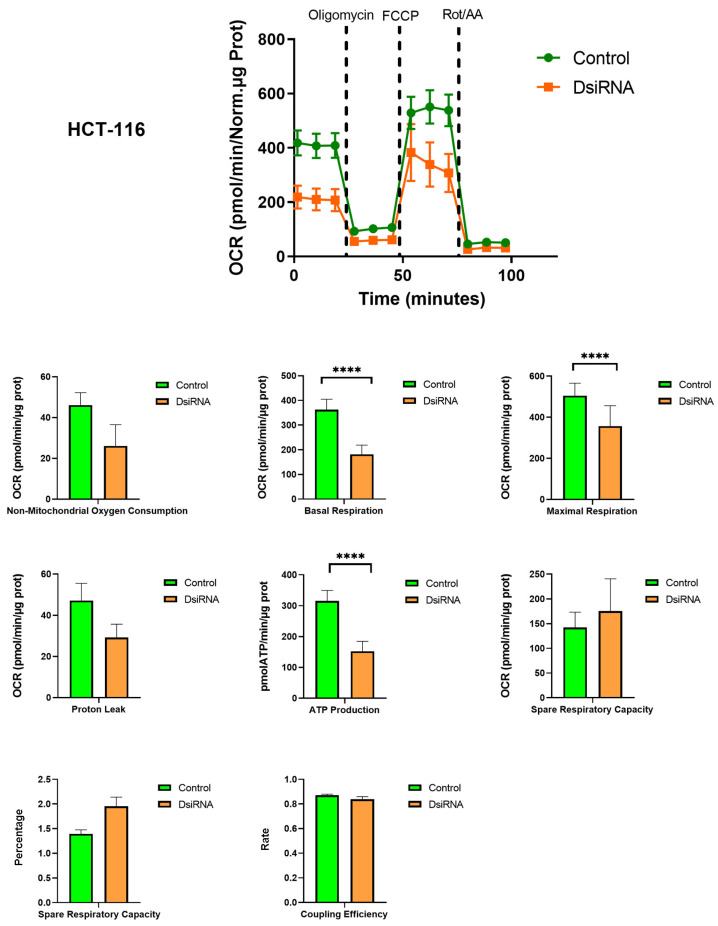
Mitochondrial metabolism in colon cancer cells HCT-116 that were exposed to DsiRNA (orange) or not (control, green), as analyzed with the Mito Stress Test on the SeaHorse platform. The upper graph represents the variations in oxygen consumption rate (OCR), depending on the application of different mitochondrial modulators. Points represent the mean ± SD of 4 experimental replicas. Histograms compare the values for control (green) and DsiRNA-treated (orange) cells for each metabolic parameter. Each bar represents the mean ± SD of 12 experimental replicas. Statistical analysis: one-way ANOVA followed by Sidak´s multiple comparisons test. ****: *p* < 0.0001.

**Figure 5:**
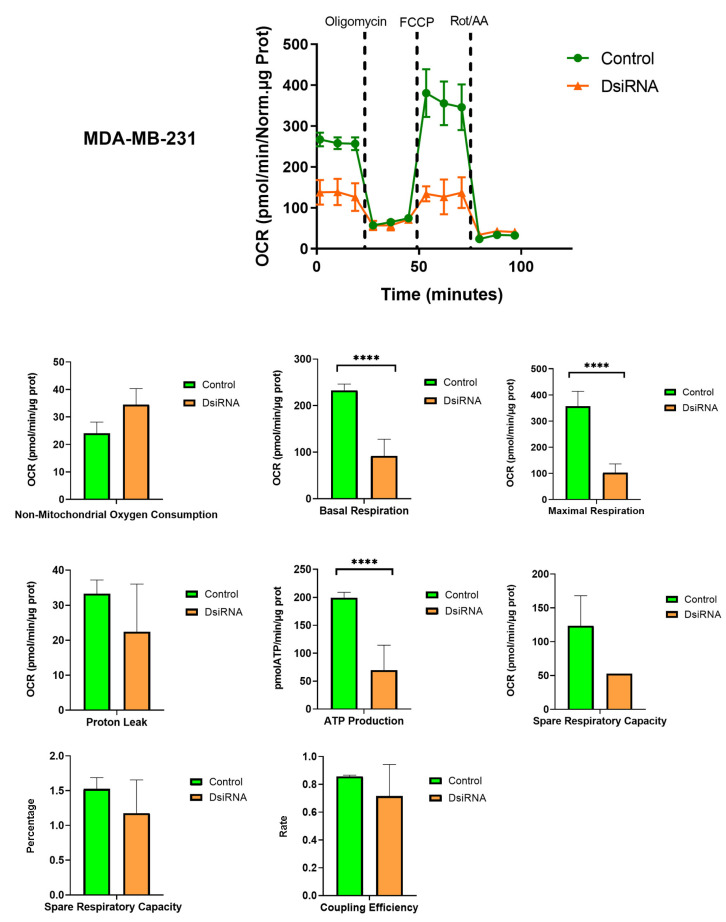
Mitochondrial metabolism in breast cancer cells MDA-MB-231 that were exposed to DsiRNA (orange) or not (control, green), as analyzed with the Mito Stress Test on the SeaHorse platform. The upper graph represents the variations in oxygen consumption rate (OCR) depending on the application of different mitochondrial modulators. Points represent the mean ± SD of 4 experimental replicas. Histograms compare the values for control (green) and DsiRNA-treated (orange) cells for each metabolic parameter. Each bar represents the mean ± SD of 12 experimental replicas. Statistical analysis: one-way ANOVA followed by Sidak´s multiple comparisons test. ****: *p* < 0.0001.

Since MDL1AS is a mitochondrial lncRNA, we wanted to study its potential impact on the main function of the mitochondria, namely, respiration. Colon and breast cancer cells, either with basal or downregulated levels of MDL1AS, were subjected to the Mito Stress Test on the SeaHorse platform. In both cell lines, we observed a very significant reduction (*p* < 0.0001) in different aspects of mitochondrial respiration, including basal respiration, maximal respiratory capacity, and ATP production, whereas no changes were found for nonmitochondrial oxygen consumption, proton leak, spare respiratory capacity, or coupling efficiency (Figure 4 and Figure 5). 

### 3.6. MDL1AS Downregulation Reduces Tumor Cell Growth in Colon Cancer Cells but Increases It in Breast Cancer Cells

**Figure 6:**
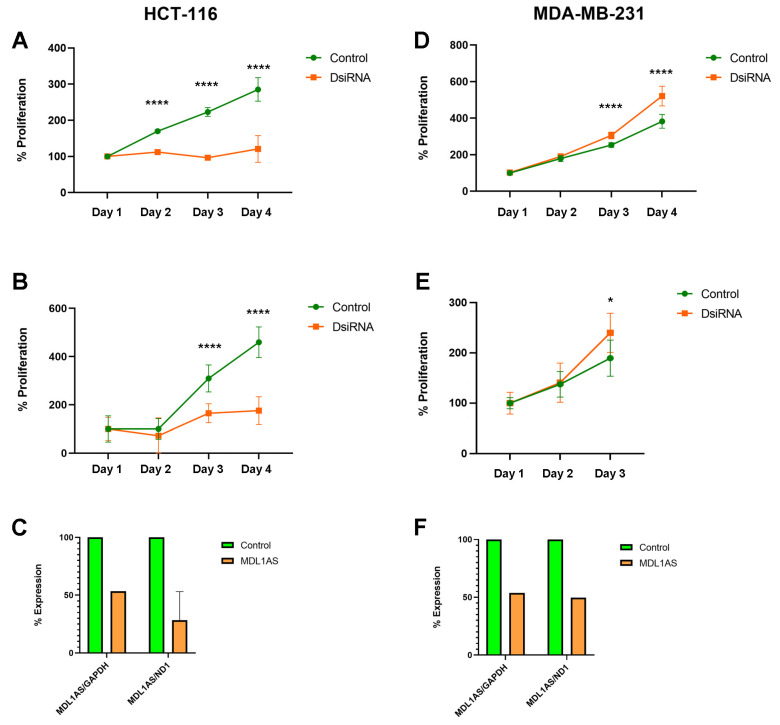
Effects of MDL1AS downregulation on proliferation potential in cell lines HCT-116 (**A**–**C**) and MDA-MB-231 (**D**–**F**). The proliferation of control cells (green) was compared with their DsiRNA-exposed counterparts (orange) using either the MTS assay (**A**,**D**) or direct cell counts (**B**,**E**). After the tests, expression of MDL1AS was always tested to confirm gene downregulation (**C**,**F**). Points represent the mean ± SD of 8 experimental replicas. Statistical analysis: one-way ANOVA followed by Sidak´s multiple comparisons test. *: *p* < 0.05; ****: *p* < 0.0001.

The MTS experiments showed a large reduction in cell number in the colon-transfected cells with lower levels of MDL1AS (*p* < 0.0001, Figure 6A). On the contrary, reduction of MDL1AS levels in breast cancer cells resulted in an increase in cell growth (*p* < 0.0001, Figure 6D). 

Since the downregulation of MDL1AS affects mitochondrial physiology (Figure 4 and Figure 5) and the MTS assay is based on the reduction of a formazan salt by mitochondrial activity [32], we used a second assay, based on direct cell counting, to confirm the impact of MDL1AS downregulation in growth. Results were very similar to those obtained with the first assay (Figure 6B,E), thus confirming the diametrically opposed behavior of both cell lines.

After the physiological experiments, the levels of MDL1AS were established by qRT-PCR, thus confirming gene downregulation (Figure 6C,F).

### 3.7. MDL1AS Downregulation Reduces Tumor Cell Migration in Colon Cancer Cells but Increases It in Breast Cancer Cells

**Figure 7:**
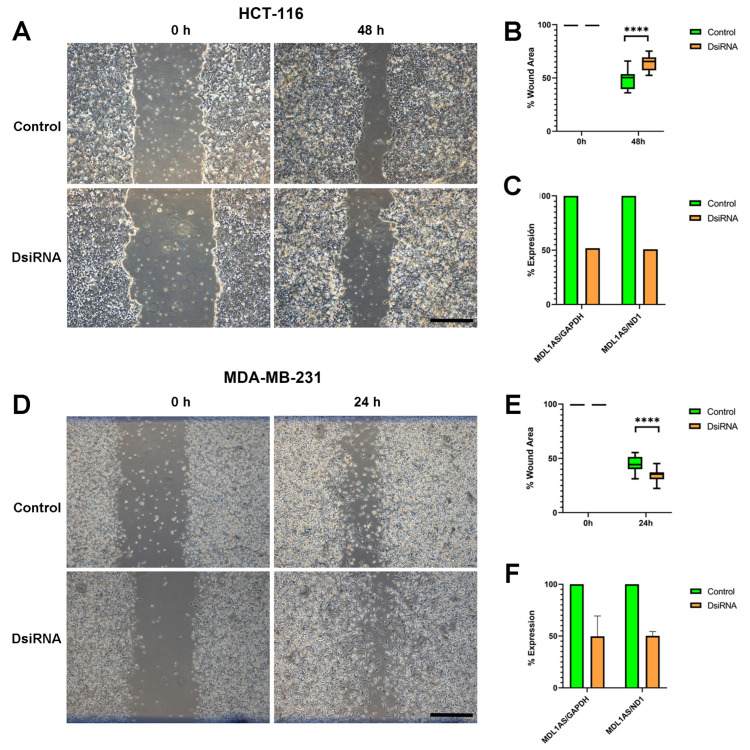
Effects of MDL1AS downregulation on migration potential in cell lines HCT-116 (**A**,**B**) and MDA-MB-231 (**D**,**E**) at the specified time points. The migration of control cells (green) was compared with their DsiRNA-exposed (orange) counterparts using the scratch assay. After the tests, expression of MDL1AS was always tested to confirm gene downregulation (**C**,**F**). Box plots represent the interquartile range with the median as the horizontal line. Whiskers encompass the maximum and minimum values of the population. Scale bars = 100 µm. Statistical analysis: one-way ANOVA followed by Sidak´s multiple comparisons test. ****: *p* < 0.0001.

Another hallmark of cancer progression is cell migration. To analyze the impact of MDL1AS on this activity, we performed a scratch experiment with cells having either basal or downregulated levels of the lncRNA. After 2 days of healing, colon cancer cells with normal levels of MDL1AS had covered more empty space than cells with lower levels of the lncRNA (*p* < 0.0001, Figure 7A–C). On the other hand, breast cancer cells were faster, closing the wound in 24 h, but, again, had the reverse behavior, with DsiRNA-transfected cells being faster in closing the wound (*p* < 0.0001, Figure 7D–F).

### 3.8. MDL1AS Downregulation Modulates Expression of Genes Related to Apoptosis and the Cell Cycle

**Figure 8:**
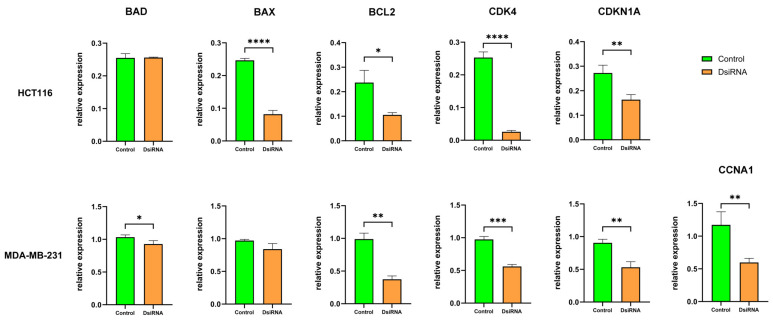
Expression of genes related to apoptosis (BAD, BAX, BCL2) or the cell cycle (CDK4, CDKN1A, CCNA1) in HCT116 (**upper row**) and MDA-MB-231 (**lower row**) cells with normal (green) or downregulated (orange) levels of MDL1AS. All data represent gene values relative to the expression of GAPDH. Bars represent the mean ± SD of 3 experimental replicas. Statistical analysis: one-way ANOVA followed by Sidak´s multiple comparisons test. *: *p* < 0.05; **: *p* < 0.01; ***: *p* < 0.001; ****: *p* < 0.0001.

To better understand the potential intracellular pathways that may be implicated in the physiological actions associated with MDL1AS downregulation, we performed qRT-PCR in colorectal and breast cancer cells (Figure 8). Most markers were significantly downregulated in cells with lower levels of MDL1AS, with the exceptions of BAD in HCT116 and BAX in MDA-MB-231, which experimented no changes. Expression of CCNA1 was undetectable in HCT116. 

## 4. Discussion

In this study, we found that the levels of the mitochondrial lncRNA, MDL1AS, can be used as a biomarker to separate tumor from normal cells in different cancers. Interestingly, MDL1AS levels are higher in tumors of the rectum or colon than in normal tissues, whereas in tumors of the breast or the larynx, they are lower. Furthermore, we found that higher MDL1 and MDL1AS levels predict longer LARC patient survival. To try to shed some light on the physiology of MDL1AS, we performed in vitro analyses on colorectal and breast cancer cells. While downregulation of MDL1AS resulted in compromised mitochondrial metabolism in both cell lines, growth and migration were affected in opposite ways, being reduced in colorectal cells with lower levels of MDL1AS and elevated in breast cancer cells. 

Many studies have previously looked for transcriptomic biomarkers in colorectal cancer [35,36], including lncRNA-based biomarkers [37], but the relevance of the expression of MDL1 and MDL1AS was never identified. This can be explained through a neglected glitch in the annotation of the human genome. Although everyone knows that mitochondria have a circular genome, such genome is currently presented as a lineal sequence interrupted precisely at the heavy strand´s replication origin. Since the sequence coding for MDL1 and MDL1AS bridges both sides of the replication origin, mapping programs cannot properly assign expression values for these genes, thus effectively hiding them in plain view. Gao et al. were the first to recognize this problem and to propose a solution by reorganizing the target mitochondrial genome [24]. In fact, they proposed the same approach to study the chloroplast, which also has a circular genome [38]. Our results show that the levels of MDL1 and/or MDL1AS may constitute effective diagnostic and/or prognostic biomarkers for a number of cancers and that transcriptomic researchers should pay attention to this region of the mitochondrial genome.

Mean survival in LARC patients treated with multimodal management, which includes adjuvant and neoadjuvant therapies, is about 80–90% at 3 years [39] and 60–65% at 5 years [40]. In our cohort, 60.9% of patients were alive 5 years after treatment, thus confirming general trends in the efficacy of these therapies. Emerging therapeutic approaches, such as targeted immune boosting therapies, non-coding RNA-based therapies, probiotics, natural products, oncolytic viral therapies, and biomarker-driven therapies, have shown promising results in preclinical and clinical studies on colorectal cancer [41]. We hope that the identification of MDL1 and MDL1AS as diagnostic and/or prognostic biomarkers in LARC and other malignancies may help in improving patient survival.

Interestingly, MDL1 and MDL1AS levels did not correlate with other parameters expected to influence overall survival, such as KRAS mutations, smoking status, the number of affected lymph nodes, or the presence of microsatellite instability [42,43]. This suggests that MDL1 and MDL1AS levels may constitute independent biomarkers that could provide high-quality information on choosing patient treatments. It is also interesting to note that we did not find any additional potential biomarker among the whole genome when studying the transcriptomic profile of this cohort, thus highlighting the strong informative power of MDL1 and MDL1AS as biomarkers. Nevertheless, the limited size of the cohort may have introduced some biases into our results, and larger groups of patients must be studied to confirm the biomarker status of these lncRNAs.

Downregulation of MDL1AS resulted in a significant reduction in several parameters of mitochondrial metabolism in both cell lines tested. This clearly indicates that MDL1AS is involved in the maintenance and activation of the mitochondrial oxidative phosphorylation cascade. Since this process is central to mitochondrial function and cellular energetic metabolism, further studies on the exact function of MDL1AS are warranted. Furthermore, inhibition of oxidative phosphorylation has been implicated in the reduction of metastases in breast cancer [44], indicating a potential application of MDL1AS inhibitors for cancer treatment.

When comparing the expression of MDL1AS in tumor cells versus the surrounding normal tissue, we found surprising results in which tumor cells of the colon and rectum had lower levels than normal-looking surrounding cells, whereas in breast and laryngeal cancers, the expression pattern was the opposite. In agreement with the clinical observations, modulation of MDL1AS in our cell lines resulted in opposing effects on growth and migration that were dependent on the organ of origin (colon vs. breast). These findings indicate that MDL1AS engages different pathways and responses, depending on the cellular context. Non-coding RNAs are well known for regulating a large number of genes rather than having specific targets [45]. In addition, mitochondrial non-coding RNAs may act directly on mitochondrial genes or travel to the nucleus and impact a large number of unrelated pathways [46], and both MDL1 and MDL1AS have been shown to translocate to the nucleus [25,26]. Furthermore, a number of small RNAs may be produced from the degradation/processing of the MDL1AS sequence [24,47]. The actions of these small RNAs will add to the pleiotropic functions of the lncRNA. lncRNAs have been also implicated in acting as sponges for endogenous RNAs, regulating miRNA decay, mediating intrachromosomal interactions, and modulating epigenetic components [48]. These epigenetic interactions include chromatin activation, which may result in transcription enhancement [49]. A more detailed molecular analysis may provide additional information on the targets and functions of MDL1AS.

In view of the different clinical and in vitro results, it is difficult to propose a single mechanism to explain how LARC patients with higher MDL1AS levels have better overall survival. On the one hand, colon and rectal tumor cells in clinical specimens express lower MDL1AS levels than the surrounding normal tissue, so we could infer that colorectal cancer patients with higher levels of the lncRNA may have a smaller tumor cell mass burden than patients with lower levels. On the other hand, colorectal cancer cells with higher expression of MDL1AS tend to grow and migrate faster and have a more efficient mitochondrial metabolism, which, in principle, points to a disadvantage for the patients. Most probably, there are additional, yet-unknown, functions modulated by MDL1AS that may explain the exact mechanism of action behind the beneficial effects of this RNA in LARC patients. A similar example of complex biology is provided by miR29-A, which acts as a tumor suppressor but is significantly elevated in the plasma of some cancer patients [50]. Further studies may offer further insight into the exact mechanism of MDL1AS on cancer pathophysiology.

In trying to understand which intracellular pathways may be involved in the discordant physiological effects found among different cell lines, we performed a gene expression study for master regulators of apoptosis and the cell cycle [51,52]. Results indicated that most of the regulators were significantly downregulated in response to MDL1AS reduction, but a clear pattern that could explain the observed cell behavior was not found. This may indicate that the effects of these lncRNAs may be more related to epigenetic changes in the cells rather than interference with the main cell pathways.

## 5. Conclusions

In conclusion, we showed the potential of MDL1 and MDL1AS expression as diagnostic and/or prognostic biomarkers in different cancers. Furthermore, we showed that MDL1AS downregulation results in reduced mitochondrial metabolism in all cells and cell-type-specific effects on growth and migration. Although larger cohorts must be analyzed to confirm the biomarker status of MDL1 and MDL1AS, our study identifies this neglected region of the mitochondrial genome as a very informative area for cancer biology that deserves further interest.

## 6. Patents

P.G., A.M.-C., and A.M. are inventors on a patent that covers the biomarker potential and clinical applications of MDL1 and MDL1AS (P202430028).

## Figures and Tables

**Table 1:** Sequence of the DsiRNAs used to downregulate MDL1AS expression.

Name	Sequence 5′ → 3′	Strand
MDL1AS.1	GUACUACAGGUGGUCAAGUAUUUAT	+
	AUAAAUACUUGACCACCUGUAGUACAU	−
MDL1AS.2	GUCGGAUACAGUUCACUUUAGCUAC	+
	GUAGCUAAAGUGAACUGUAUCCGACAU	−
MDL1AS.3	GACAUUCAAUUGUUAUUAUUAUGTC	+
	GACAUAAUAAUAACAAUUGAAUGUCUG	−

**Table 2:** Sequence of the primers used for qRT-PCR.

	Gene	Forward 5′ → 3′	Reverse 5′ → 3′
1	*MDL1*	TCAACTGCAACTCCAAAGCC	GGGGACGAGAAGGGATTTGA
2	*MDL1*	CAGCCACTTTCCACACAGAC	GGTTAGGCTGGTGTTAGGGT
3	*MDL1AS*	CATGGGGACGAGAAGGGATT	CACACATCAACTGCAACTCCA
4	*MDL1AS*	GGTTAGGCTGGTGTTAGGGT	CAGCCACTTTCCACACAGAC
5	*MDL1AS*	ACATTACTGCCAGCCACCAT	TGCTTGTAAGCATGGGGAGG
6	*MDL1AS*	GTCCCTTGACCACCATCCTC	GGGGAACGTGTGGGCTATTT
7	*BCL2*	ATGTGTGTGGAGAGCGTCAA	GGAGGAAGTCCAATGTCCAG
8	*BAX*	GTGGCAGCTGACATGTTTTC	GGAGGAAGTCCAATGTCCAG
9	*BAD*	CGGAGGATGAGTGACGAGTT	CCAGGACTGGAAGACTCGC
10	*CCNA1*	TGAAATAAGGCACAGACCCAAAGC	ACCAGCCAGTCCACCAGAATCGT
11	*CDKN1A*	TGTCCGTCAGAACCCATGC	AAAGTCGAAGTTCCATCGCTC
12	*CDK4*	TCGTGAGGTGGCTTTACTGAGGCG	TCCTTGATCGTTTCGGCTGGCA
13	*ND1*	CCTCCTACTCCTCATTGTACCC	CAGCGAAGGGTTGTAGTAGC
14	*GAPDH*	AAATCCCATCACCATCTTCC	GACTCCACGACGTACTCAGC

**Table 3:** Statistical analysis comparing groups classified by 5-year survival.

Factor	Survival ≥ 5 Years	Survival < 5 Years	*p* Value
Age (mean ± sd)	60.47 ± 10.57	64.70 ± 9.29	0.094
Sex (Male: Female, n)	28:14	21:6	0.327
Lymph node affectation y/n	8/34	9/17	0.154
Removed lymph nodes (mean ± sd)	0.93 ± 1.27	0.81 ± 1.95	0.550
Positive lymph nodes (mean ± sd)	8.71 ± 5.02	7.96 ± 4.86	0.208
Chemotherapy n (%)	31 (73.8%)	19 (70.4%)	0.774
Mutated KRAS n (%)	9 (21.4%)	7 (26.9%)	0.610
Microsatellite instability n (%)	6 (14.6%)	3 (11.5%)	0.722
Smoker n (%)	22 (52.4%)	16 (59.3%)	0.581
MDL1 RPKM (mean ± sd)	2279.34 ± 1080.24	1976.40 ± 596.34	0.187
MDL1AS RPKM (mean ± sd)	1630.60 ± 875.62	1278.23 ± 387.18	0.053

**Table 4:** Patient characteristics when classified into high and low expressors of MDL1AS (cut-off = 1980 RPKM).

Factor	High Expression	Low Expression	*p* Value
Age (mean ± sd)	64.93 ± 8.40	61.35 ± 10.62	0.233
Sex (Male: Female, n)	10:5	39:15	0.680
Lymph node affectation y/n	6/9	11/42	0.132
Removed lymph nodes (mean ± sd)	0.93 ± 1.27	0.81 ± 1.95	0.813
Positive lymph nodes (mean ± sd)	8.71 ± 4.69	8.35 ± 5.05	0.820
Chemotherapy n (%)	12 (80%)	38 (70%)	0.507
Mutated KRAS n (%)	2 (14%)	14 (26%)	0.368
Microsatellite instability n (%)	3 (21%)	6 (11%)	0.331
Smoker n (%)	6 (40%)	32 (59%)	0.189
MDL1 RPKM (mean ± sd)	3426.88 ± 863.44	1809.11 ± 573.59	<0.00001
MDL1AS RPKM (mean ± sd)	2580.66 ± 736.16	1190.51 ± 366.02	<0.00001

## Data Availability

Sequencing data from the patient cohort are available at NCBI as BioProject PRJNA1071368 (http://www.ncbi.nlm.nih.gov/bioproject/1071368 (accessed on 26 February 2024)).
